# 4.6 Å Cryo-EM reconstruction of tobacco mosaic virus from images recorded at 300 keV on a 4k × 4k CCD camera

**DOI:** 10.1016/j.jsb.2010.06.011

**Published:** 2010-09

**Authors:** Daniel K. Clare, Elena V. Orlova

**Affiliations:** Crystallography, Department of Biological Sciences, Birkbeck College, University of London, Malet Street, London WC1E 7HX, United Kingdom

**Keywords:** TMV, Cryo-EM, Helical processing, Single particle processing and CCD camera

## Abstract

Tobacco mosaic virus (TMV) is a plant virus with a highly ordered organisation and has been described in three different structural states: As stacked disks without RNA (X-ray crystallography), as a helical form with RNA (X-ray fibre diffraction) and as a second distinct helical form with RNA (cryo-EM). Here we present a structural analysis of TMV as a test object to assess the quality of cryo-EM images recorded at 300 keV on a CCD camera. The 4.6 Å TMV structure obtained is consistent with the previous cryo-EM structure and confirms that there is a second helical form of TMV. The structure here also shows that with a similar number of TMV segments an equivalent resolution can be achieved with a 4k CCD camera at 300 keV.

## 1 Introduction

Cryo-electron microscopy (Cryo-EM) has become a well established technique for the structural determination of biological macromolecules to sub-nanometre resolution. In recent years the resolution achievable by single particle cryo-EM has improved due to modernisation in both data collection and image processing. Data quality has been improved by the use of more stable stages, field emission guns and automated procedures used to collect very large data sets (Jiang et al., 2008; Yu et al., 2008; Zhang et al., 2008a, 2009; Sachse et al., 2007; Ludtke et al., 2008; Stagg et al., 2006, 2008; Suloway et al., 2005). The development of image processing methodologies allows heterogeneous populations to be separated and single particle procedures to be applied to homogeneous sub-populations (Elad et al., 2007, 2008; Zhang et al., 2008b; Scheres et al., 2007).

Digital recording of images has become a standard method in cryo-EM labs. CCD cameras have been shown to have a higher signal-to-noise ratio (SNR) at low-to-intermediate resolutions (25–10 Å) than photographic film but a lower SNR at high resolutions (beyond 10 Å) (Sander et al., 2005; Booth et al., 2004; Faruqi and Henderson, 2007). However, two recent studies have shown that the SNR even at high resolutions (around 5 Å) are the same for images recorded at 300 or 120 keV either on film or CCD camera if the correct imaging conditions are used (Booth et al., 2006; Mooney, 2007). One of the benefits of using high-voltage electrons is related to decreasing both elastic and inelastic scattering cross sections allowing the imaging of thicker specimens. High-voltage electrons are also closer to the single-scattering approximation and thus the image is a more accurate representation of the biological object. The electron-optical design of high-voltage microscopes has improved, giving better coherence and brightness of the electron beam (Downing and Mooney, 2008). All these factors combined give better high resolution information at higher electron voltages. A problem with high-voltage electrons is that they get scattered in the scintillator of the CCD camera and therefore generate multiple photons, causing resolution loss and increased noise in the recorded image (Downing and Hendrickson, 1999; Booth et al., 2004; Faruqi and Henderson, 2007). However, it has been shown that higher-energy electrons can be detected without strong scattering in the scintillator if a more dense material, such as cadmium telluride (CdTe) or gallium arsenide (GaAs) are used instead of silicon (McMullan et al., 2007). In this model experiment they demonstrated that the lateral spread of electrons in CdTe at 300 keV is less than that at 200 keV in silicon.

An advantage of CCD cameras is that at low electron doses the signal transfer is more linear than that of film which provides a more correct representation of the beam intensity (Booth et al., 2004, 2006; Sander et al., 2005; Chen et al., 2008). This improves the SNR at low-to-intermediate resolutions on CCD images versus film which should help in the alignment (Sander et al., 2005). One drawback of CCD cameras is the relatively large size of the sensor element that is required for trustworthy registration of the signal (15–24 μm) when compared to that of film densitometers (5–7 μm). However, this problem can be overcome by increasing of the magnification by two-to-three times when recording images on CCD camera versus film. A consequence of large size sensor size and higher magnifications used is a restricted field of view of CCD images (a 16k × 16k chip has been developed but most laboratories do not have cameras greater than 4k × 4k). The small field of view limits the number of particles per image but this can be compensated for by collecting more images which can preferably be done using one of the automated data collection packages such as AutoScan, Leginon and JADAS (Zhang et al., 2003, 2009; Suloway et al., 2005).

In this study we used TMV to test whether a near atomic resolution structure can be obtained using a commercially available 4k × 4k CCD camera with an ultra sensitive scintillator to collect cryo-EM images at 300 keV. TMV is a highly ordered plant virus. Mature TMV is approximately 3000 Å in length and 180 Å in width, with a 40 Å central channel, and consists of RNA and multiple copies of a 17.5 kDa coat protein (CP). It has been found in different conformational states by X-ray crystallography at 2.5 Å (stacked disks conformation), X-ray fibre structure at 2.9 Å (helical conformation) and cryo-EM at 4.7 Å (second distinct helical conformation) (Bhyravbhatla et al., 1998; Namba et al., 1989; Sachse et al., 2007). The CP consists mainly of α-helices arranged perpendicular to the helical axis. The CP can be split into three regions: the high radius (HR) region (furthest from the centre of the virus), the middle radius (MR) region and the low radius (LR) region (close to the centre of the virus). The main differences between the three solved structures seem to occur in the LR region (Sachse et al., 2007). This region contains key resides involved in RNA binding (Arg 90 and 92) and in the assembly/disassembly of the virus (Glu 95, 97,106 and Asp109). The interaction between Arg92-RNA was only observed in the cryo-EM structure and not in the fibre structure, and seems to induce more order in the LR region. A pair of carboxylates in the MR (Glu50 and Asp77) has also been shown to affect assembly/disassembly of TMV (Culver et al., 1995).

The resulting EM density map of TMV at 4.6 Å demonstrates clearly that near atomic resolution structure is feasible for non-icosahedral particles at 300 keV with a 4k CCD camera. This structure is consistent with the previous cryo-EM structure, showing the same conformation in the LR region and a similar resolution.

## 2 Results and discussion

**Fig. 1:**
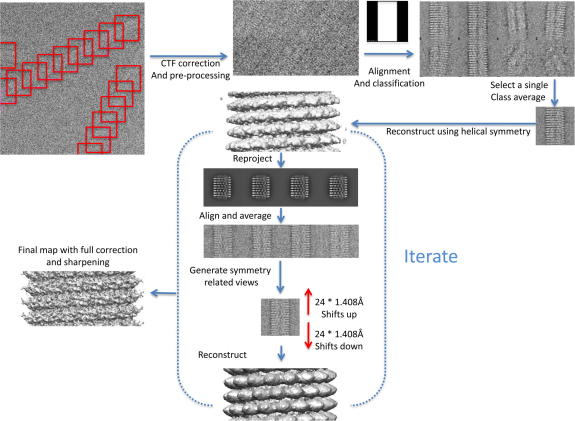
Image processing scheme. A schematic of the image processing steps used to reconstruct the helical form of TMV.

We started our analysis by extracting 5300 TMV segments from 104 manually collected CCD frames, equivalent to 260 000 asymmetric subunits (ASU). In order to reduce noise the segments were initially 2 × 2 averaged to give 2.48 Å/pixel at the specimen level. The magnification was determined using the computed diffraction patterns of the TMV images and known periodicity of the virus. The image processing was done using a similar method to that described by Sachse et al. (2007). Individual TMV virus particles were cut into segments, CTF corrected and aligned (see Section 3). The aligned segments with the same angle assignment were averaged and assuming their known helical parameters symmetry-related views were generated. The symmetry-related views were then used to calculate a reconstruction which was used to generate a new set of references images for further rounds of alignment (Procedure summarised in Fig. 1). In TMV 49 CP subunits lead to three turns, with a height of 69 Å and a helical rise per subunit of 1.408 Å and a rotation per subunit of 22.04° (equivalent to the ASU) (Holmes and Franklin, 1958; Namba et al., 1989). The main difference in our approach from that of Sachse and co-workers is that we used shorter segments and that the initial three-dimensional (3D) reconstruction was generated from a single class average and its symmetry-related views (see Section 3, Fig. 1).

**Fig. 2:**
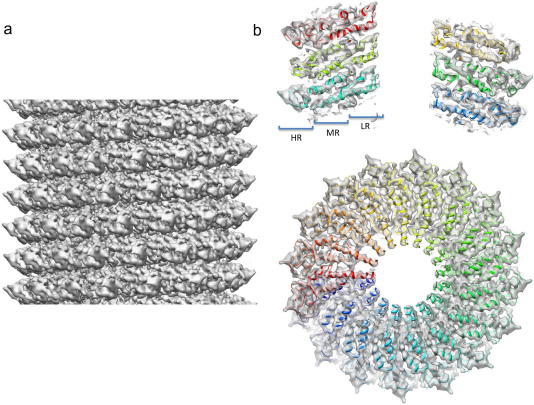
Helical reconstruction of TMV. The fully corrected sharpened reconstruction from 260 000 ASU of TMV, shown from the side (a), as a central axial-section (b), and in cross-section. The docked atomic coordinates of the 2OM3 structure are shown in cartoon representation (rainbow coloured).

With the coarsened sampling, the Fourier shell correlation (FSC) of the reconstruction, extended almost to the Nyquist limit (∼5 Å) (Supplementary Fig. 1). Therefore we decided to process the un-binned images, which have a sampling of 1.24 Å/pixel, thus increasing the achievable resolution but also the noise level. For the final reconstruction, amplitude and phase CTF correction was performed and the map was sharpened (see Section 3). The resolution of the final reconstruction was estimated by FSC to be 4.6 Å using the 0.5 criterion (Supplementary Fig. 1b). Atomic coordinates 2TMV (X-ray fibre structure) and 20M3 (cryo-EM structure) were docked into the cryo-EM density using Chimera (Goddard et al., 2007). Using visual inspection and cross-correlation coefficients (the cc value was 14% lower for 2TMV compared to 2OM3) as guides it was obvious that 2OM3, the atomic coordinates generated from the previous cryo-EM study, gave a better fit to the density (Fig. 2; only 2OM3 fit shown; for a comparison of the X-ray fibre structure and the previous cryo-EM structure see Sachse et al., 2007; Supplementary Fig. 2). This suggests that our TMV structure represents the second distinct helical conformation seen previously by cryo-EM. The 3_10_ helix, Arg92-RNA contact and a similar inner loop conformation are seen in both cryo-EM maps (Fig. 2; Supplementary Fig. 2). However, we noted some small differences in the inner channel-lining loop, which has the weakest density, and in the position and orientation of some side chain residues (Supplementary Fig. 2). In order to analyse this we used the X-ray crystallographic programme Coot (Emsley and Cowtan, 2004) to modify the 2OM3 structure using a combination of real space refinement (allowing all atoms to move), rigid body refinement (moving a single or multiple residues as one rigid body) and structure regularisation (to correct peptide bond/side chain geometry and apply Ramachandran restraints). After this remodelling the RMSD between 2OM3 and our coordinates was 0.743 Å.

**Fig. 3:**
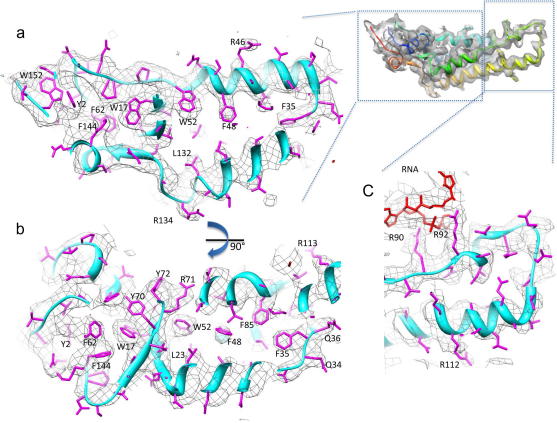
The refined atomic coordinates of a TMV monomer fitted into the map. Side view (a) and top view (b) of the HR region of the refined monomer fitted into the density map of TMV contoured at 2*σ*. Side view (c) of the LR region of the refined monomer fitted into the density map of TMV contoured at 1*σ*. The LR region was contoured at a lower threshold as the inner channel-lining loop disappears at higher thresholds, probably due to greater conformational flexibility.

The fit of the refined structure into the cryo-EM density map showed more of the side chains and the main chain of the inner loop were in density (Fig. 3, Supplementary Fig. 3). Inspection of the fit reveals the same characteristic side chain density distribution as that observed by Sachse and co-workers, including well defined density for nearly all the large aromatic residues (Phe, Tyr and Trp) nearly all nitrogen containing side chains (Arg and Gln) but very few carboxylates (Glu and Asp) (Supplementary Fig. 4). It is also clear that there is no side chain density or noise around the acidic residues. The lack of density for Glu and Asp residues has been reported in previous cryo-EM studies. It has been suggested that carboxyl groups are particularly susceptible to radiation damage (Grigorieff et al., 1996; Mitsuoka et al., 1999; Sachse et al., 2007).

The small structural differences between our map and the previous cryo-EM study (overall RMSD of 0.743 Å) may be explained by differences in sample conditions: TMV structure is known to be sensitive to ionic conditions and temperature (Pattanayek and Stubbs, 1992). In this study we used the same pH as the previous cryo-EM study, but lower concentrations of KCl and MgCl_2_ and the sample was maintained at room temperature. The sample in the previous study had no salt, included EDTA and was maintained at 4 °C. However, the observed differences are small for this helical conformation of TMV.

In conclusion our results demonstrate that it is possible to reveal side chains in structures determined form cryo-EM images collected at 300 keV on a 4k × 4k standard CCD camera used at full resolution, using single particle analysis with helical symmetry. This is also the first non-ichoshedral virus structure that it has been possible to resolve side chains using a CCD camera. We have also confirmed that the second helical form of TMV previously reported by cryo-EM is the solution state of the virus. Our results corroborate that the use of commonly produced cameras (while depending on the quality of the sample, state of a microscope and skills of a researcher) open a road to high-throughput in EM structural analysis.

## 3 Methods

### 3.1 Protein expression and purification

The TMV was provided by Dr. Jan W.M. van Lent from the Laboratory of Virology, Wageningen, Netherlands and was purified and assembled as described by Gooding and co-workers (Gooding and Hebert, 1967).

### 3.2 Sample preparation and electron microscopy

TMV was diluted to a final concentration of 3 mg/ml in 50 mM Tris–HCl (pH 7.4), 50 mM KCl, 10 mM MgCl_2_. 3.5 μl of the TMV was applied to C-flat grids (r2/2, Protochips Inc., USA), which had been rendered hydrophilic by glow discharge in air. The grids were then blotted and frozen in liquid nitrogen cooled liquid ethane.

Low dose images (104 images, 20–25 e^−^/Å^2^) were manually recorded from two grids on a Tecnai Polara electron microscope (FEI, Netherlands) operated at 300 keV, using a Gatan Ultrascan 4000 4k × 4 k CCD camera with an ultra sensitive phosphor scintillator (Gatan, USA). A calibrated magnification of 121 000× and a defocus range between 0.9 and 3.0 μm underfocus were used during data collection. Magnification was calibrated using the first layer line from power spectra of multiple TMV particles. The four quadrants of the CCD camera had been equalised and the images were gain normalised and dark corrected.

### 3.3 Image processing

Five thousand and three hundred segments of the TMV particles were picked using the helical option in the EMAN programme Boxer (Ludtke et al., 1999). The overlap between boxes was set so that there were 4–5 unique turns of TMV per cut out 300 pixel box. The contrast transfer function (CTF) for each CCD image was determined using CTFFIND3 (Mindell and Grigorieff, 2003). CTF phase correction was done in SPIDER, and the images were then band-pass filtered between 200 and 3 Å and normalised. Segments were aligned using a soft edged rectangle reference and then classified by multi-variate statistical analysis (MSA) in IMAGIC (van Heel et al., 1996). The best class average was used as a reference for a further round of alignment followed by classification using MSA. The helical rise of 1.408 Å was then used to shift the best class average along the y axis to generate the 48 symmetry-related views.

The 49 views were assigned angles starting from 0° in steps of 22.04°. A reconstruction was then calculated, using the BP RP command in SPIDER, and used as a reference for projection matching. Initially the structure was reprojected in the range of angles 80–100° with respect to the helical axis and 0–22.04° around the helical axis with a step size of 2°, which gave 102 reference images. After a three rounds of alignment and reconstruction the step size was decreased to 1° giving 427 reference images (ran 7 further rounds of alignment). After each round of alignment the images that had aligned to the same reference were averaged and their symmetry-related views calculated using TMV’s helical parameters:

$$\begin{array}{cc} & y_{i}=y_{\text{o}}+i\Delta y_{\text{a}}\\ & r_{j}=r_{\text{o}}+i\Delta r_{\text{a}} \end{array}$$

where *y*_o_ = the centre of the image, *r*_o_ = the assigned angle around the symmetry axis; −24 ≤ *i* ≤ 24; Δ*y*_a_ = 1.408 Å and Δ*r*_a_ = 22.04°.

Once the angle assignment had stabilised the resolution of the map was estimated by the Fourier shell correlation (FSC) and found to be 4.6 Å at the 0.5 FSC criterion. The two reconstruction used in the FSC calculation were generated separately by splitting the final average and symmetry-related views into even and odd numbers. In the final reconstruction the full CTF correction was done in SPIDER to correct for the envelope function of the microscope. An envelope parameter of 0.25 was used in SPIDER (Frank et al., 1996) to model the amplitude fall-off of the CTF as it was similar to the observed fall-off in the experimental images. The individual images were multiplied by their corresponding CTF function, band-pass filtered and normalised as before. The alignment determined for each segment was then applied and they were averaged (as above) and this average was then divided by the sum of the CTF functions squared plus a Wiener filter value of 0.1 to complete the full CTF correction. The fully corrected averages were then shifted by the helical rise to generate the symmetry-related views and used to reconstruct TMV. The map was sharpened using a smooth edged filter that suppressed frequency components lower than 10 Å by 85% and removed high frequency components beyond 4 Å. The central part of the sharpened reconstruction that comprised three turns of the TMV helix was subjected to the helical symmetrisation to generate the averaged TMV map. As a final refinement step this map was then used to align the multiplied images with an angular step of 0.5° and a limited angular range (3° from the already assigned angle). The aligned images were then treated as described above. The only exception was that instead of applying a smoothed edge filter to sharpen the map we used the programme EMBFACTOR (Fernandez et al., 2008) to estimate the B-factor and apply the inverse of that B-factor to sharpen the map. The B-factor was estimated to be 155 Å^2^ between 10 and 4 Å and therefore −155 Å^2^ was used to scale amplitudes. The combination of a finer angular spacing and the use of the B-factor improved the EM density so that both the main chain and side chain densities were better resolved.

### 3.4 Atomic structure fitting and refinement

The 2TMV and 2OM3 structures were docked into the final map using Chimera (Goddard et al., 2007). It was evident that the cryo-EM structure was closer to that of 2OM3 but it needed some further refinement of the LR region and some side chains. The cross-correlation between 2TMV, 2OM3 and our modified coordinates with our cryo-EM map were determined in Chimera using a resolution cutoff of 3.5 Å and were 0.39, 0.46 and 0.51, respectively. The structure was refined with Coot (Emsley and Cowtan, 2004), using geometry analysis and the Ramachandran plot to validate the structure. Once the structure fulfilled both criteria it was again docked into the map with Chimera, and showed an improved cross-correlation with the cryo-EM density. The Ramachandran plot showed that 94.74% of residues were in the preferred region with only 5.26% in the allowed region.

### 3.5 Accession codes

Both the cryo-EM density map and the atomic coordinates have been deposited in the EMDB and PDB with accession codes EMD-1730 and 2XEA.

## References

[bib1] Bhyravbhatla B., Watowich S.J., Caspar D.L. (1998). Refined atomic model of the four-layer aggregate of the tobacco mosaic virus coat protein at 2.4-Å resolution. Biophys. J..

[bib2] Booth C.R., Jiang W., Baker M.L., Zhou Z.H., Ludtke S.J., Chiu W. (2004). A 9 angstroms single particle reconstruction from CCD captured images on a 200 kV electron cryomicroscope. J. Struct. Biol..

[bib3] Booth C.R., Jakana J., Chiu W. (2006). Assessing the capabilities of a 4 × 4 k CCD camera for electron cryo-microscopy at 300 kV. J. Struct. Biol..

[bib4] Chen D.H., Jakana J., Liu X., Schmid M.F., Chiu W. (2008). Achievable resolution from images of biological specimens acquired from a 4 × 4 k CCD camera in a 300-KV electron cryomicroscope. J. Struct. Biol..

[bib5] Culver J.N., Dawson W.O., Plonk K., Stubbs G. (1995). Site-directed mutagenesis confirms the involvement of carboxylate groups in the disassembly of tobacco mosaic virus. Virology.

[bib6] Downing K.H., Hendrickson F.M. (1999). Performance of a 2 k CCD camera designed for electron crystallography at 400 kV. Ultramicroscopy.

[bib7] Downing K.H., Mooney P.E. (2008). A charge coupled device camera with electron decelerator for intermediate voltage electron microscopy. Rev. Sci. Instrum..

[bib8] Elad N., Farr G.W., Clare D.K., Orlova E.V., Horwich A.L., Saibil H.R. (2007). Topologies of a substrate protein bound to the chaperonin GroEL. Mol. Cell.

[bib9] Elad N., Clare D.K., Saibil H.R., Orlova E.V. (2008). Detection and separation of heterogeneity in molecular complexes by statistical analysis of their two-dimensional projections. J. Struct. Biol..

[bib10] Emsley P., Cowtan K. (2004). Coot: model-building tools for molecular graphics. Acta Crystallogr. D Biol. Crystallogr..

[bib11] Faruqi A.R., Henderson R. (2007). Electronic detectors for electron microscopy. Curr. Opin. Struct. Biol..

[bib12] Fernandez J.J., Luque D., Caston J.R., Carrascosa J.L. (2008). Sharpening high resolution information in single particle electron cryomicroscopy. J. Struct. Biol..

[bib13] Frank J., Radermacher M., Penczek P., Zhu J., Li Y., Ladjadj M., Leith A. (1996). SPIDER and WEB: processing and visualization of images in 3D electron microscopy and related fields. J. Struct. Biol..

[bib14] Goddard T.D., Huang C.C., Ferrin T.E. (2007). Visualizing density maps with UCSF Chimera. J. Struct. Biol..

[bib15] Gooding G.V., Hebert T.T. (1967). A simple technique for purification of tobacco mosaic virus in large quantities. Phytopathology.

[bib16] Grigorieff N., Ceska T.A., Downing K.H., Baldwin J.M., Henderson R. (1996). Electron-crystallographic refinement of the structure of bacteriorhodopsin. J. Mol. Biol..

[bib17] Holmes K.C., Franklin R.E. (1958). The radial density distribution in some strains of tobacco mosaic virus. Virology.

[bib18] Jiang W., Baker M.L., Jakana J., Weigele P.R., King J., Chiu W. (2008). Backbone structure of the infectious epsilon15 virus capsid revealed by electron cryomicroscopy. Nature.

[bib19] Ludtke S.J., Baldwin P.R., Chiu W. (1999). EMAN: semiautomated software for high-resolution single-particle reconstructions. J. Struct. Biol..

[bib20] Ludtke S.J., Baker M.L., Chen D.H., Song J.L., Chuang D.T., Chiu W. (2008). De novo backbone trace of GroEL from single particle electron cryomicroscopy. Structure.

[bib21] McMullan G., Cattermole D.M., Chen S., Henderson R., Llopart X., Summerfield C., Tlustos L., Faruqi A.R. (2007). Electron imaging with Medipix2 hybrid pixel detector. Ultramicroscopy.

[bib22] Mindell J.A., Grigorieff N. (2003). Accurate determination of local defocus and specimen tilt in electron microscopy. J. Struct. Biol..

[bib23] Mitsuoka K., Hirai T., Murata K., Miyazawa A., Kidera A., Kimura Y., Fujiyoshi Y. (1999). The structure of bacteriorhodopsin at 3.0 Å resolution based on electron crystallography: implication of the charge distribution. J. Mol. Biol..

[bib24] Mooney P. (2007). Optimization of image collection for cellular electron microscopy. Methods Cell Biol..

[bib25] Namba K., Pattanayek R., Stubbs G. (1989). Visualization of protein–nucleic acid interactions in a virus. Refined structure of intact tobacco mosaic virus at 2.9 Å resolution by X-ray fiber diffraction. J. Mol. Biol..

[bib26] Pattanayek R., Stubbs G. (1992). Structure of the U2 strain of tobacco mosaic virus refined at 3.5 Å resolution using X-ray fiber diffraction. J. Mol. Biol..

[bib27] Sachse C., Chen J.Z., Coureux P.D., Stroupe M.E., Fandrich M., Grigorieff N. (2007). High-resolution electron microscopy of helical specimens: a fresh look at tobacco mosaic virus. J. Mol. Biol..

[bib28] Sander B., Golas M.M., Stark H. (2005). Advantages of CCD detectors for de novo three-dimensional structure determination in single-particle electron microscopy. J. Struct. Biol..

[bib29] Scheres S.H., Gao H., Valle M., Herman G.T., Eggermont P.P., Frank J., Carazo J.M. (2007). Disentangling conformational states of macromolecules in 3D-EM through likelihood optimization. Nat. Methods.

[bib30] Stagg S.M., Lander G.C., Pulokas J., Fellmann D., Cheng A., Quispe J.D., Mallick S.P., Avila R.M., Carragher B., Potter C.S. (2006). Automated cryoEM data acquisition and analysis of 284 742 particles of GroEL. J. Struct. Biol..

[bib31] Stagg S.M., Lander G.C., Quispe J., Voss N.R., Cheng A., Bradlow H., Bradlow S., Carragher B., Potter C.S. (2008). A test-bed for optimizing high-resolution single particle reconstructions. J. Struct. Biol..

[bib32] Suloway C., Pulokas J., Fellmann D., Cheng A., Guerra F., Quispe J., Stagg S., Potter C.S., Carragher B. (2005). Automated molecular microscopy: the new Leginon system. J. Struct. Biol..

[bib33] Van Heel M., Harauz G., Orlova E.V., Schmidt R., Schatz M. (1996). A new generation of the IMAGIC image processing system. J. Struct. Biol..

[bib34] Yu X., Jin L., Zhou Z.H. (2008). 3.88 Å structure of cytoplasmic polyhedrosis virus by cryo-electron microscopy. Nature.

[bib35] Zhang P., Borgnia M.J., Mooney P.E., Shi D., Pan M., Herron P.O., Mao A., Brogan D., Milne J.L.S., Subramaniam S. (2003). Automated image acquisition and processing using a new generation of 4 × 4 k CCD cameras for cryo electron microscopic studies of macromolecular assemblies. J. Struct. Biol..

[bib36] Zhang X., Settembre E., Xu C., Dormitzer P.R., Bellamy R., Harrison S.C., Grigorieff N. (2008). Near-atomic resolution using electron cryomicroscopy and single-particle reconstruction. Proc. Natl. Acad. Sci. USA.

[bib37] Zhang W., Kimmel M., Spahn C.M., Penczek P.A. (2008). Heterogeneity of large macromolecular complexes revealed by 3D cryo-EM variance analysis. Structure.

[bib38] Zhang J., Nakamura N., Shimizu Y., Liang N., Liu X., Jakana J., Marsh M.P., Booth C.R., Shinkawa T., Nakata M., Chiu W. (2009). JADAS: a customizable automated data acquisition system and its application to ice-embedded single particles. J. Struct. Biol..

