# Supplementary material for: 4.6 Å Cryo-EM reconstruction of tobacco mosaic virus from images recorded at 300 keV on a 4k × 4k CCD camera
Source: J Struct Biol. 2010 Sep;171(3-3):303–8. doi: 10.1016/j.jsb.2010.06.011 (PMC2939825; doi:10.1016/j.jsb.2010.06.011)
Supplement: Supplementary data — Supplementary Figures. [file mmc1.pdf]

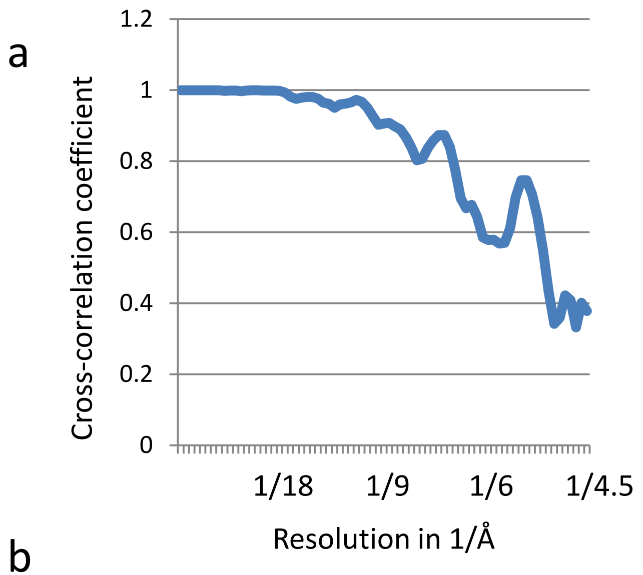

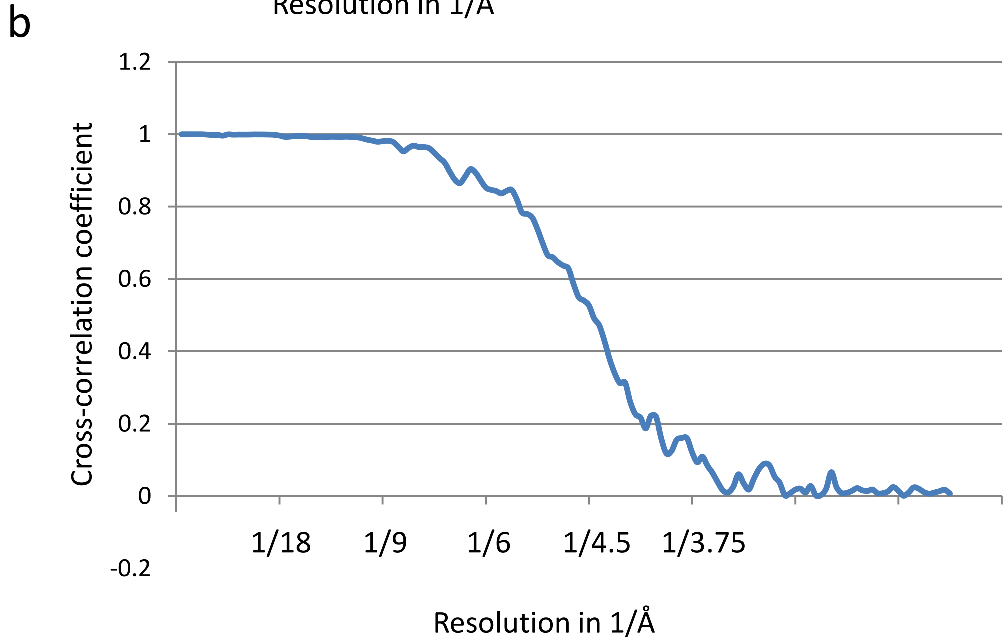

Supplementary Figure 1.

Fourier shell correlations

The Fourier shell correlation from two reconstructions of the data set at a sampling of 2.48 Å/pixel (a). Fourier shell correlation from two reconstructions of the 260,000 ASU data set at a sampling of 1.24 Å/pixel (b).

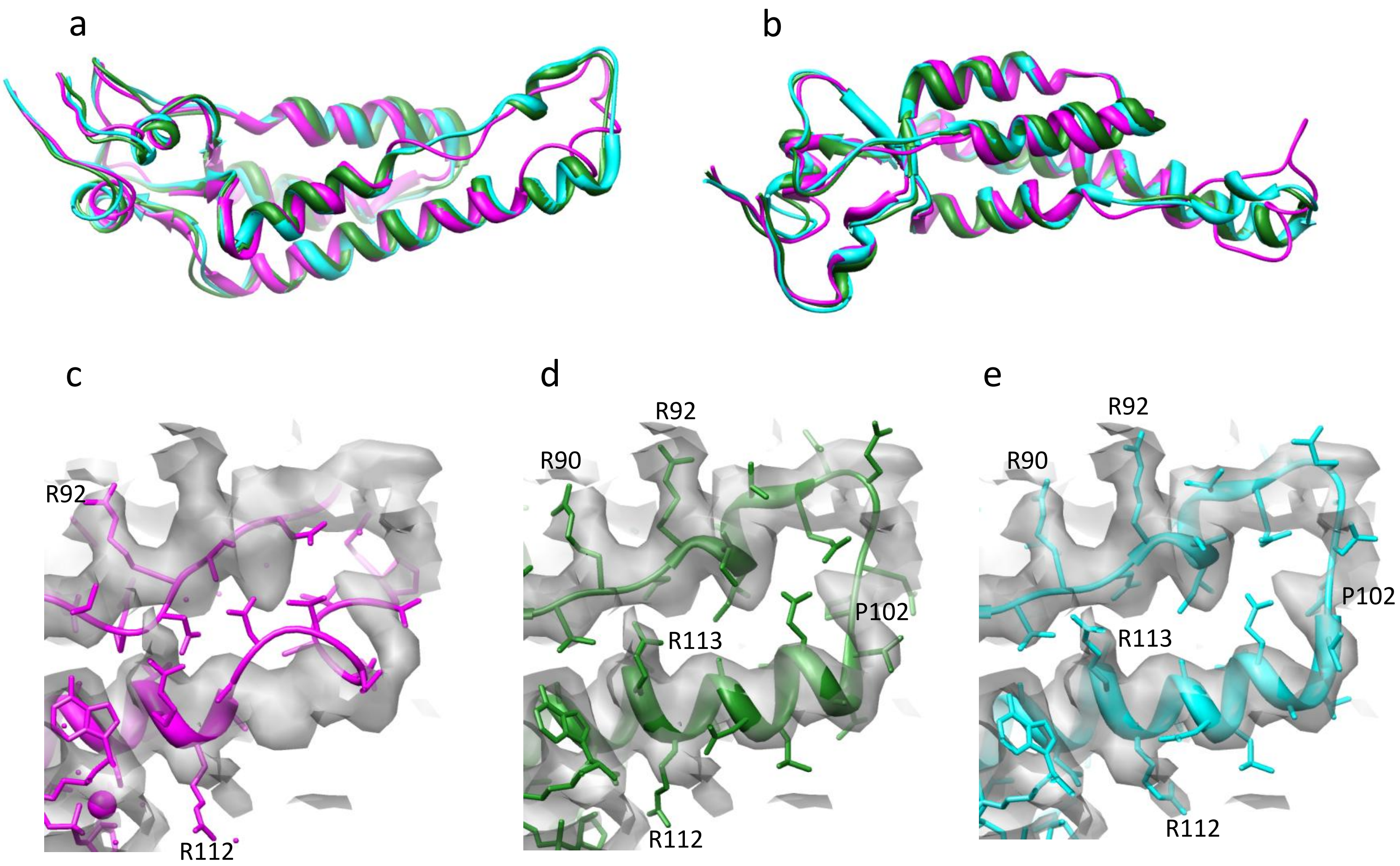

Supplementary Figure 2. **Comparison of the different TMV structures**
The c-alpha coordinates for 2TMV (magenta), 2OM3 (green) and our fit 2XEA (blue) are shown from the side (a) and from the top (b). The coordinates for the 2TMV, 2OM3 and 2XEA are shown with the cryo-EM density focusing on the region of most diversity between each of the structures (c, d and e). This comparison shows the main difference between the 2TMV and the two cryo-EM derived structures. The differences between our structure and 2OM3 are in the inner loop position and some changes in side chain orientations (R90, R112, and P102).

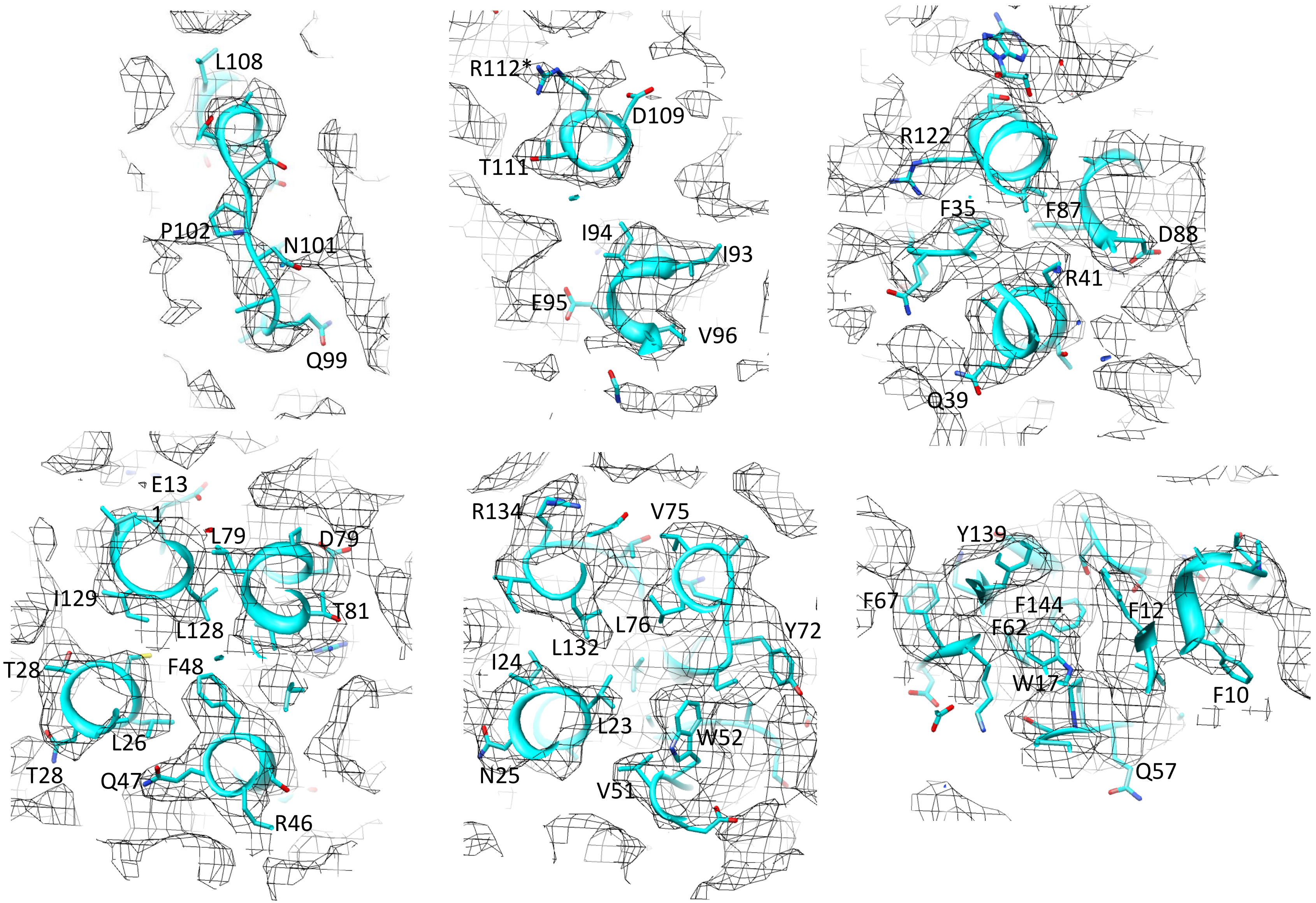

Supplementary Figure 3. **Cross-sections of the EM map and fitted coordinates**

Cross-section through the cryo-EM with the fitted atomic model shown from the inside the TMV cylinder starting from the inner loop of the CP (top left) and running through to the hydrophobic core (bottom right). The density is displayed as mesh at 1σ and a number of residues throughout the structure have been labelled.

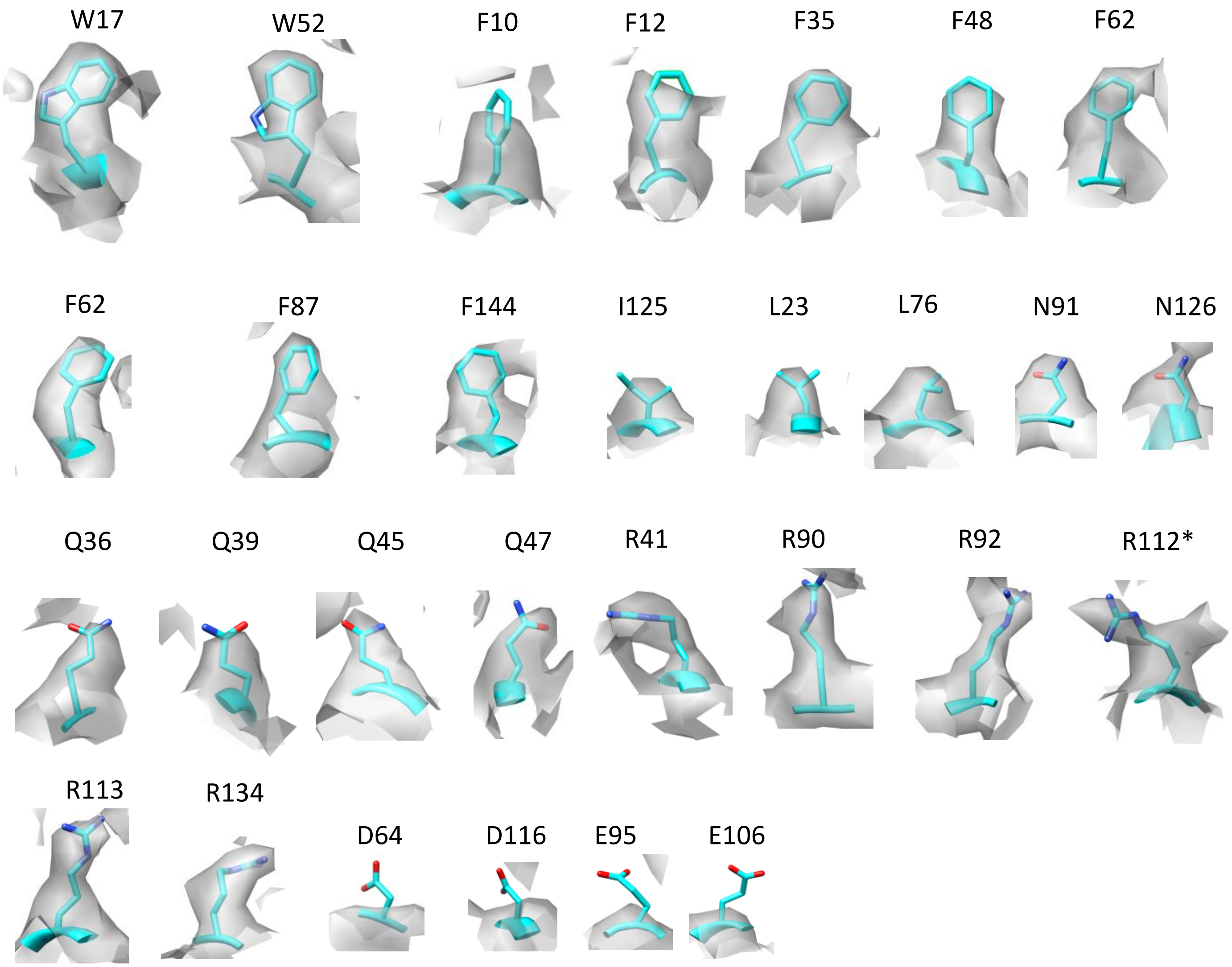

Supplementary Figure 4. **Side chain densities**

A montage of fitted side chains in their corresponding density. From this it is clear that the density for the large hydrophobics, some of the small hydrophobics and the basic side chains are in agreement with the fitted coordinates.
